# Formalin-Fixed and Paraffin-Embedded Samples for Next Generation Sequencing: Problems and Solutions

**DOI:** 10.3390/genes12101472

**Published:** 2021-09-23

**Authors:** Gerardo Cazzato, Concetta Caporusso, Francesca Arezzo, Antonietta Cimmino, Anna Colagrande, Vera Loizzi, Gennaro Cormio, Teresa Lettini, Eugenio Maiorano, Vincenza Sara Scarcella, Paola Tarantino, Maricla Marrone, Alessandra Stellacci, Paola Parente, Paolo Romita, Aurora De Marco, Vincenzo Venerito, Caterina Foti, Giuseppe Ingravallo, Roberta Rossi, Leonardo Resta

**Affiliations:** 1 Section of Molecular Pathology, Department of Emergency and Organ Transplantation (DETO), University of Bari “Aldo Moro”, 70124 Bari, Italy; k.caporusso@libero.it (C.C.); micasucci@inwind.it (A.C.); anna.colagrande@gmail.com (A.C.); lettinit@yahoo.com (T.L.); eugenio.maiorano@uniba.it (E.M.); vincenza.scarcella@policlinico.ba.it (V.S.S.); tarpa80@gmail.com (P.T.); Roberta.rossi@policlinico.ba.it (R.R.); leonardo.resta@uniba.it (L.R.); 2 Section of Ginecology and Obstetrics, Department of Biomedical Science and Oncology (DIMO), University of Bari “Aldo Moro”, 70124 Bari, Italy; francesca.arezzo@uniba.it (F.A.); vera.loizzi@uniba.it (V.L.); gennaro.cormio@uniba.it (G.C.); 3 Section of Legal Medicine, Interdisciplinary Department of Medicine, Bari Policlinico Hospital, University of Bari “Aldo Moro”, Piazza Giulio Cesare 11, 70124 Bari, Italy; mariclamarrone@hotmail.it (M.M.); alestellacci@gmail.com (A.S.); 4 UOC di Anatomia Patologica, Fondazione IRCCS Casa Sollievo Della Sofferenza, 71013 San Giovanni Rotondo, Italy; paolaparente77@gmail.com; 5 Section of Dermatology, Department of Biomedical Sciences and Human Oncology, University of Bari “Aldo Moro”, Piazza Giulio Cesare 11, 70124 Bari, Italy; paolo.romita@uniba.it (P.R.); aurorademarco94@gmail.com (A.D.M.); caterina.foti@uniba.it (C.F.); 6 Section of Reumathology, Department of Emergency and Organ Transplantation (DETO), University of Bari “Aldo Moro”, 70124 Bari, Italy; vincenzo.venerito@policlinico.ba.it

**Keywords:** NGS, FFPE, PCR, DNA, RNA

## Abstract

Over the years, increasing information has been asked of the pathologist: we have moved from a purely morphological diagnosis to biomolecular and genetic studies, which have made it possible to implement the use of molecular targeted therapies, such as anti-epidermal growth factor receptor (EGFR) molecules in EGFR-mutated lung cancer, for example. Today, next generation sequencing (NGS) has changed the approach to neoplasms, to the extent that, in a short time, it has gained a place of absolute importance and diagnostic, prognostic and therapeutic utility. In this scenario, formaldehyde-fixed and paraffin-embedded (FFPE) biological tissue samples are a source of clinical and molecular information. However, problems can arise in the genetic material (DNA and RNA) for use in NGS due to fixation, and work is being devoted to possible strategies to reduce its effects. In this paper, we discuss the applications of FFPE tissue samples in the execution of NGS, we focus on the problems arising with the use of this type of material for nucleic acid extraction and, finally, we consider the most useful strategies to prevent and reduce single nucleotide polymorphisms (SNV) and other fixation artifacts.

## 1. Introduction

In recent decades, a complete change in diagnostic techniques has occurred in various sectors of medicine. Among these, next generation sequencing (NGS) [1] is certainly gaining an increasingly important role, to the extent that we have moved on from only morphological characterization of tumors, although this is still fundamental, to a more detailed and extensive analysis of a constantly increasing number of genes [2]. Indeed, the latest frontier is to achieve the personalization of drugs exclusively on the basis of gene profiling (so-called agnostic therapy) without necessarily paying attention to the tissue from which the sample [3] was obtained. Of course, there is no shortage of more than justified skepticism about this development, but the issue offers evidence of the growing faith, perhaps exaggerated, but certainly current, in NGS. As technical aspects are being perfected, this technique is becoming ever faster and more efficient [4]. In this perspective, tissues fixed in formaldehyde and included in paraffin (FFPE) have a role of absolute importance. The possibility of conducting advanced molecular biology investigations on previously acquired material may transform (as is already happening in some parts of the world) Pathological Anatomy Laboratory archives into real mines of information. In this paper, we focus on these aspects, taking as reference the most recent discoveries in the scientific field, and discuss the limitations regarding the use of FFPE for NGS that still exist, while taking a look at possible scenarios in the near and distant future.

## 2. Materials and Methods

A systematic review was conducted following the Preferred Reporting Items for Systematic Reviews and Meta-Analyses (PRISMA) guidelines. A search of PubMed and Web of Sciences (WoS) databases was performed using the terms: “formalin-fixed paraffin-embedded (FFPE) tissues” OR “formalin-fixed tissues” OR “paraffin-embedded tissues” AND/OR “next generation sequencing (NGS)” OR “gene profiling” OR “DNA extraction ” OR “DNA-seq” OR “RNA-seq”. Only articles in English were selected. Eligible articles were assessed according to the Oxford Centre for Evidence-Based Medicine 2011 guidelines [5]. Review articles, meta-analyses and observational studies were included. Other potentially relevant articles were identified by manually checking the references of the included literature.

An independent extraction of articles was performed by two investigators according to the inclusion criteria. Disagreement was resolved by discussion between the two review authors. We focused on describing the molecular problems of FFPE, their relation to NGS, and other artifact problems, and discuss possible approaches to optimize these analyses.

## 3. Results

**Figure 1:**
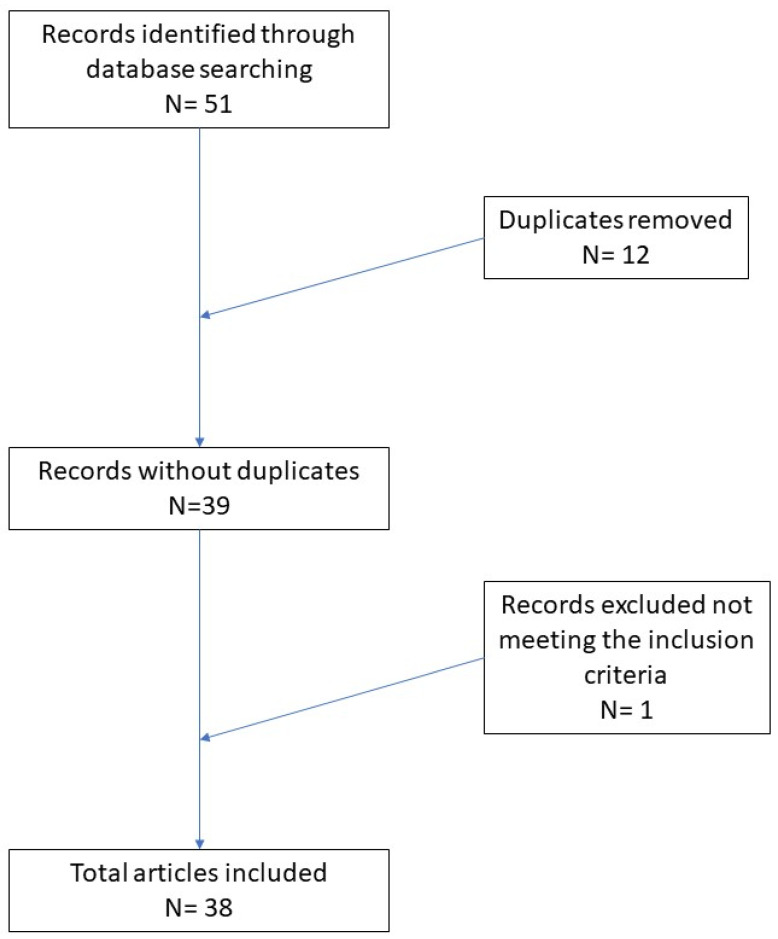
Literature search and article selection.

In total, 51 records were initially identified in the literature search, 12 of which were duplicates. After screening for eligibility and inclusion criteria, 38 publications were ultimately included (Figure 1). The study and characteristics are summarized in Supplementary Table S1. The publications included above all reviews (*n* = 19), followed by comparative studies (*n* = 9), original articles (*n* = 4), clinical trials (*n* = 3), and meta-analyses (*n* = 3). All studies included were rated as level 4 or 5 evidence for clinical research, as detailed in the Oxford Centre for Evidence-Based Medicine 2011 guidelines [5].

## 4. Discussion

Formaldehyde (FA) is the fixative par excellence for biological material in Pathology [6,7], despite its recognized toxic and carcinogenic properties [8,9]. It is now a consolidated technique to use buffered formaldehyde at 10% or 20% as it is important to maintain a neutral pH: only at restricted pH levels can nucleic acids (DNA and RNA) be kept in such conditions as to allow the execution of biomolecular investigations [10]. The ever-increasing developments in NGS have focused attention on the possible suitability of formaldehyde-fixed and paraffin-embedded tissues for molecular investigations, as well as for immunohistochemical investigations only [11]. Although formaldehyde can preserve tissue morphology and properties, various works have dealt with the problems that arise from formaldehyde-protein-nucleic acid contact. In detail, formaldehyde can affect the DNA double helix, sometimes severely damaging the quality of the DNA used after FFPE. In fact, FA can interact with different DNA structures through the formation of crosslinks, which can occur in different points: histone–DNA crosslinks, formaldehyde–DNA adducts, DNA–protein crosslinks, and DNA–DNA crosslinks [12,13,14,15,16,17]. Moreover, after FFPE the DNA is more subject to certain modifications such as the deamination of cytosine and 5′methyl-cytosine, forming uracil and thiamine. Recognized by the DNA polymerase, these can also cause sequence artifacts like C:G > T:A, C:G > A:T, C:G > G:C, A:T > G:C, or else an abasic site may form, which weakens the intrinsic structure of the DNA double helix, or may even rupture it [13]. All of these reactions can potentially alter the correct sequences during the later processes, such as polymerase chain reaction (PCR) and NGS. As early as 1998, Williams et al. [18] demonstrated that the rate of mutations present in FFPE tissues was higher than in matched frozen samples. These authors cautioned against using FFPE material without an adequate knowledge of the rate of possible mutations, which was up to 1 mutation per 500 bp. Furthermore, the problem related to the lack of recognition of “artificial” mutations from FFPE tissues was pointed out. These would be incorporated into mutation banks of the tumor under study, raising the risk of impeding a correct genetic-molecular analysis [18].

In 2004, Quach et al. described an increased number of spontaneous mutations in FFPE tissues subjected to PCR. In detail, they described a 3/4-fold higher percentage of mutations in fixed tissues compared to fresh frozen tissues and demonstrated that the use of Taq DNA polymerase could reduce these sequence artifacts arising through translation synthesis subject to errors [19]. Other studies have confirmed that multiple artifact sequence alterations, for example in the EGFR gene, arise in FFPE lung tissues [20]. Gallegos Ruiz et al. evaluated 47 cases of tumor tissue from patients affected by non-small cell lung cancer (NSCLC) both FFPE and fresh frozen, extracting both genomic DNA (gDNA) and RNA for the determination of any alterations of EGFR-R and K-ras. PCR was successful in 100% of fresh frozen tissue cases, while it was successful in only 50% of FFPE tissue cases. In fact, the authors found new EGFR mutations (insertions/deletions) in DNA from both FFPE and fresh frozen tissue but the rate of artifactual mutations was very low when RNA was isolated from fresh frozen tissue as compared to FFPE tissue. This led to the conclusion that fresh tissue RNA was more reliable for molecular analysis [20]. A systematic review of 3381 somatic EGFR mutations detected in 12,244 patients with non-small cell lung cancer found that 71% of the EGFR mutations were seen in only a single case, suggesting that many of the reported EGFR mutations may be sequence artifacts [21].

Tsao et al. reported several new EGFR mutations in FFPE DNA [22], that had never been found in over 2000 fresh frozen non-small cell lung cancer specimens [23]. Similar findings have been reported in other studies that highlighted the possibility of sequence artifacts on FFPE samples [24,25]. Ofner et al. presented their data related to the analysis of 96 melanoma samples, which revealed a total of 46 ERBB4 mutations in 27 samples, including the identification of 11 mutations in three previously unknown mutational hotspots. The authors, unable to confirm any presumed hotspot mutations within the repeated sequencing of relevant amplicons, concluded that they were most likely sequence artifacts due to FFPE [26]. Högnäs et al. compared the methods of preparing fresh frozen, formalin-fixed and paraffin-embedded (FFPE) and PAX gene-fixed paraffin-embedded (PFPE) tissues in prostate radical prostatectomy tissue from 36 patients and performed a preliminary feasibility test of the use of tissue PFPE in the routine diagnostic evaluation of surgical prostate disease. Their findings suggest that where DNA and/or RNA tissue analysis is required, and when the tissue size is small, PFPE can provide important advantages over FFPE. They made a brief review of what measures may be adopted to improve the yield of DNA and RNA from FFPE [27]. Pérez-Báez et al. [28] reported on 104 cases of colorectal cancer analyzed by high resolution melting analysis (HRMA) for KRAS mutation detection and found a high rate of sequence artifacts of the material from FFPE samples, confirming reports by many other authors [25,26,28,29,30,31]. Sah et al. [29], starting from the assumption that FFPE tissue cannot always provide good quality DNA, conducted a study with a new PCR technique, called “QFI-PCR”, applied to 165 tissue samples. This technique allows quantification, in an absolute sense, of the number of copies of DNA that can really be amplified, demonstrating that adequate quantities of genetic material are fundamental for the subsequent application of NGS.

## 5. Strategies for Minimization of Sequence Artifacts from FFPE DNA

One of the most critical points in order to achieve satisfactory NGS analysis is the choice of the sample. An expert pathologist needs to select the blocks containing a representative sample of the tumor to be studied, in order to allow a minimum quantity of material (not less than 20%) from which to undertake the subsequent biomolecular analyses [12,29,32]. Furthermore, the risk of breakage of the cross-links between the various molecules caused by formaldehyde increases with thermal pre-treatment: it has been shown that the use of a temperature > 90° can lead to reversibility of the cross-links formed. Thus, to ensure the quality of the subsequent PCR amplification reactions, it is important to start from an adequate base of nucleic acids [30,31,32,33]. A possible strategy to minimize the sequence artifacts that lead to single nucleotide variants (SNV) is to pretreat the material with uracil-DNA-glycosylase, an enzyme that can recognize uracil formed by cytosine deamination and cleave it from the DNA chain, generating an abasic site. Most of the DNA polymerases used, not being able to bypass the abasic site, will remain blocked, preventing the sequence artifact from being repeated [34]. Indeed, Do and Dobrovic. A. have demonstrated that pretreatment with uracil-DNA glycosylase before the PCR reaction is able to drastically reduce the sequence artifacts (mainly constituted by non-reproducible C:G > T:A substitutions). In their experiment, the authors employed the high resolution fusion (HRM) technique to evaluate the presence of any mutations, and aimed to ascertain whether the genetic material from FFPE was altered from the beginning of the analysis. In addition to confirming these data, already known in the literature, the authors added a new step immediately before the PCR amplification: they added UDG to the solution, which statistically significantly reduced the presence of uracil instead of deaminated cytosine. In this way, they prevented an amplification error from occurring. Furthermore, the pretreatment did not affect the detection of “true” mutations such as the KRAS codon 12 mutation and EGFR exon 19 and 20 mutations [34,35]. In 2019, McDonough, S.J et al. published their paper on the use of nine methods of extracting DNA from FFPE tissues. The authors used twelve FFPE samples from different tissue types, monitoring quality indicators such as total yield, percentage of dsDNA, fragment analysis and multiplex PCR. After the first evaluation, they selected three tissue types from four FFPE DNA methods for downstream evaluation of NGS, targeted and whole exome sequencing. Additionally, two low-input library protocols for WES were evaluated. The results revealed that the mean coverage across target regions for WES was ~20–30× for all four FFPE DNA extraction methods. For the targeted panels, the highest molecular tag coverage was achieved with the Kingfisher FFPE extraction method. Genotype agreement was 99% for positions commonly called variants between all four extraction methods with the targeted NGS PCR panel and 96% with WES. Therefore, the authors concluded that assessing the quality of the extracted DNA helps to select the optimal NGS approach, and the choice of DNA extraction and library preparation approaches can affect the performance of archival tissue in NGS [36].

Also in 2019, Bhagwate et al. conducted an elegant experiment in a pilot study, analyzing paired FFPE-derived DNA samples and fresh frozen breast tissues for FFPE-specific artifacts. For FFPE samples, they used two FFPE DNA extraction methods to determine the impact of wet lab procedures on calling the variants: the QIAGEN QIAamp DNA Mini Kit (“QA”) and QIAGEN GeneRead DNA FFPE Kit (“QGR”). All DNA sample libraries were prepared for NGS according to the QIAseq Human Breast Cancer Targeted DNA Panel protocol and sequenced on the HiSeq 4000. The authors performed detailed variant concordance comparisons and mutational signature analysis to study the effects of FFPE samples versus fresh frozen paired samples, along with different DNA extraction methods. Among the various findings, they showed that five-fold or more variants were called with FFPE samples, compared to paired fresh frozen tissue samples, even after applying molecular barcode error correction and the default bioinformatics filter recommended by the supplier. Furthermore, as an optimized approach for FFPE-DNA extraction, QGR leads to far fewer discordant variants between fresh frozen paired samples and FFPE. Approximately 92% of the uniquely named FFPE variants had a low allelic frequency range (<5%) and collectively shared a “C > T|G > A” mutational signature known to be representative of FFPE artifacts resulting from cytosine deamination. This study demonstrated the feasibility of calling and filtering genetic variants from FFPE tissue samples using a combined strategy with molecular barcodes, optimized DNA extraction, and bioinformatics methods that incorporate the genomic context such as the mutational signature and variant allelic frequency [32,37].

## 6. Conclusions and Perspectives

From the studies examined in our work, although these constitute only a fraction of the information available in the literature, it is quite clear that the advent of NGS has renewed interest in molecular information obtainable from FFPE tissue. It is clear, however, that several problems are encountered when carrying out NGS investigations of the nucleic acids extracted from this type of tissue. This has already prompted researchers to study alternative, new ways to reduce formaldehyde fixation artifacts/mutations, in order to maximize the quantity and quality of information obtainable from FFPE. New studies, experiments and techniques for the extraction of nucleic acids will increase the reliability of these resources, that are now becoming essential in the pathological anatomy and precision oncology field [38,39].

## Data Availability

Not applicable.

## Supplementary Materials

The following are available online at https://www.mdpi.com/article/10.3390/genes12101472/s1, Table S1: Most significant studies present in literature.

## References

[1] Aly S., Sabri D.M. (2015). Next generation sequencing (NGS): A golden tool in forensic toolkit. Arch. Forensic Med. Criminol..

[2] Liu L., Li Y., Li S., Hu N., He Y., Pong R., Lin D., Lu L., Law M. (2012). Comparison of Next-Generation Sequencing Systems. J. Biomed. Biotechnol..

[3] Lavacchi D., Roviello G., D’Angelo A. (2020). Tumor-Agnostic Treatment for Cancer: When How is Better than Where. Clin. Drug Investig..

[4] Pareek C.S., Smoczynski R., Tretyn A. (2011). Sequencing technologies and genome sequencing. J. Appl. Genet..

[5] Oxford Centre for Evidence-Based Medicine 2011 Levels of Evidence. http://www.cebm.net/wp-content/uploads/2014/06/CEBM-Levels-of-Evidence-2.1.pdf.

[6] Loomis T.A. (1979). Formaldehyde toxicity. Arch. Pathol. Lab. Med..

[7] Farooqui M.Y. (1983). Formaldehyde. J. Appl Toxicol..

[8] Nilsson J.A., Heng X., Sundqvist K., Liu Y., Atzori L., Elfwing A., Arvidson K., Grafström R.C. (1998). Toxicity of formaldehyde to human oral fibroblasts and epithelial cells: Influences of culture conditions and role of thiol status. J. Dent. Res..

[9] Kwak K., Paek D., Park J. (2020). Occupational exposure to formaldehyde and risk of lung cancer: A systematic review and meta-analysis. Am. J. Ind. Med..

[10] Plénat F., Antunes L., Haller T., Piet-Ounnoughene M., Klein-Monhoven N., Champigneulle J., Chenal P., Bland V., Garcia-Pimenta F., Labouyrie E. (2001). Formaldehyde fixation in the third millennium. Ann. de Pathol..

[11] Xuan J., Yu Y., Qing T., Guo L., Shi L. (2012). Next-generation sequencing in the clinic: Promises and challenges. Cancer Lett..

[12] Do H., Dobrovic A. (2015). Sequence Artifacts in DNA from Formalin-Fixed Tissues: Causes and Strategies for Minimization. Clin. Chem..

[13] Schweiger M.R., Kerick M., Timmermann B., Albrecht M.W., Borodina T., Parkhomchuk D., Zatloukal K., Lehrach H. (2009). Genome-Wide Massively Parallel Sequencing of Formaldehyde Fixed-Paraffin Embedded (FFPE) Tumor Tissues for Copy-Number- and Mutation-Analysis. PLoS ONE.

[14] Feldman M.Y. (1973). Reactions of nucleic acids and nucleoproteins with formaldehyde. Prog. Nucleic Acid Res. Mol. Biol..

[15] Fraenkel-Conrat H., Olcott H.S. (1948). The Reaction of Formaldehyde with Proteins. V. Cross-linking between Amino and Primary Amide or Guanidyl Groups. J. Am. Chem. Soc..

[16] McGhee J.D., von Hippel P.H. (1975). Formaldehyde as a probe of DNA structure. II. Reaction with endocyclic imino groups of DNA bases. Biochemistry.

[17] Ludyga N., Grünwald B., Azimzadeh O., Englert S., Höfler H., Tapio S., Aubele M. (2012). Nucleic acids from long-term preserved FFPE tissues are suitable for downstream analyses. Virchows Arch..

[18] Williams C., Pontén F., Moberg C., Söderkvist P., Uhlen M., Pontén J., Sitbon G., Lundeberg J. (1999). A High Frequency of Sequence Alterations Is Due to Formalin Fixation of Archival Specimens. Am. J. Pathol..

[19] Quach N., Goodman M.F., Shibata D. (2004). In vitro mutation artifacts after formalin fixation and error prone translesion synthesis during PCR. BMC Clin. Pathol..

[20] Ruiz M.I.G., Floor K., Rijmen F., Grünberg K., A Rodriguez J., Giaccone G. (2007). EGFR and K-ras Mutation Analysis in Non-Small Cell Lung Cancer: Comparison of Paraffin Embedded versus Frozen Specimens. Cell Oncol..

[21] Murray S., Dahabreh I.J., Linardou H., Manoloukos M., Bafaloukos D., Kosmidis P. (2008). Somatic Mutations of the Tyrosine Kinase Domain of Epidermal Growth Factor Receptor and Tyrosine Kinase Inhibitor Response to TKIs in Non-small Cell Lung Cancer: An Analytical Database. J. Thorac. Oncol..

[22] Tsao M., Sakurada A., Cutz J.-C., Zhu C., Kamel-Reid S., Squire J., Lorimer I., Zhang T., Liu N., Daneshmand M. (2005). Erlotinib in Lung Cancer—Molecular and Clinical Predictors of Outcome. N. Engl. J. Med..

[23] Didelot A., Kotsopoulos S.K., Lupo A., Pekin D., Li X., Atochin I., Srinivasan P., Zhong Q., Olson J., Link D.R. (2013). Multiplex Picoliter-Droplet Digital PCR for Quantitative Assessment of DNA Integrity in Clinical Samples. Clin. Chem..

[24] Suzuki T., Ohsumi S., Makino K. (1994). Mechanistic studies on depurination and apurinic site chain breakage in oligodeoxyribonucleotides. Nucleic Acids Res..

[25] Zsikla V., Baumann M., Cathomas G. (2004). Effect of buffered formalin on amplification of DNA from paraffin wax embedded small biopsies using real-time PCR. J. Clin. Pathol..

[26] Ofner R., Ritter C., Ugurel S., Cerroni L., Stiller M., Bogenrieder T., Solca F., Schrama D., Becker J.C. (2017). Non-reproducible sequence artifacts in FFPE tissue: An experience report. J. Cancer Res. Clin Oncol..

[27] Högnäs G., Kivinummi K., Kallio H.M.L., Hieta R., Ruusuvuori P., Koskenalho A., Kesseli J., Tammela T.L.J., Riikonen J., Ilvesaro J. (2018). Feasibility of Prostate PAXgene Fixation for Molecular Research and Diagnostic Surgical Pathology: Comparison of Matched Fresh Frozen, FFPE, and PFPE Tissues. Am. J. Surg. Pathol..

[28] Pérez-Báez W., García-Latorre E.A., Maldonado-Martínez H.A., Coronado-Martínez I., Flores-García L., Taja-Chayeb L. (2017). Impact of fixation artifacts and threshold selection on high resolution melting analysis for KRAS mutation screening. Mol. Cell. Probes.

[29] Sah S., Chen L., Houghton J., Kemppainen J., Marko A.C., Zeigler R., Latham G.J. (2013). Functional DNA quantification guides accurate next-generation sequencing mutation detection in formalin-fixed, paraffin-embedded tumor biopsies. Genome Med..

[30] Jackson V. (1978). Studies on histone organization in the nucleosome using formaldehyde as a reversible cross-linking agent. Cell.

[31] Shi S.-R., Cote R.J., Wu L., Liu C., Datar R., Shi Y., Liu D., Lim H., Taylor C.R. (2002). DNA Extraction from Archival Formalin-fixed, Paraffin-embedded Tissue Sections Based on the Antigen Retrieval Principle: Heating Under the Influence of pH. J. Histochem. Cytochem..

[32] Campos P.F., Gilbert T.M.P. (2011). DNA Extraction from Formalin-Fixed Material. Methods Mol. Biol..

[33] Wu L., Patten N., Yamashiro C.T., Chui B. (2002). Extraction and Amplification of DNA From Formalin-Fixed, Paraffin-Embedded Tissues. Appl. Immunohistochem. Mol. Morphol..

[34] Do H., Dobrovic A. (2012). Dramatic reduction of sequence artefacts from DNA isolated from formalin-fixed cancer biopsies by treatment with uracil-DNA glycosylase. Oncotarget.

[35] Do H., Wong S.Q., Li J., Dobrovic A. (2013). Reducing Sequence Artifacts in Amplicon-Based Massively Parallel Sequencing of Formalin-Fixed Paraffin-Embedded DNA by Enzymatic Depletion of Uracil-Containing Templates. Clin. Chem..

[36] McDonough S.J., Bhagwate A., Sun Z., Wang C., Zschunke M., Gorman J.A., Kopp K.J., Cunningham J.M. (2019). Use of FFPE-derived DNA in next generation sequencing: DNA extraction methods. PLoS ONE.

[37] Bhagwate A.V., Liu Y., Winham S.J., McDonough S.J., Stallings-Mann M.L., Heinzen E.P., Davila J.I., Vierkant R.A., Hoskin T.L., Frost M. (2019). Bioinformatics and DNA-extraction strategies to reliably detect genetic variants from FFPE breast tissue samples. BMC Genom..

[38] Kresse S.H., Namløs H.M., Lorenz S., Berner J.-M., Myklebost O., Bjerkehagen B., Meza-Zepeda L.A. (2018). Evaluation of commercial DNA and RNA extraction methods for high-throughput sequencing of FFPE samples. PLoS ONE.

[39] Mullegama S.V., Alberti M.O., Au C., Li Y., Toy T., Tomasian V., Xian R.R. (2018). Nucleic Acid Extraction from Human Biological Samples. Methods Mol. Biol..

