# Supplementary material for: Formalin-Fixed and Paraffin-Embedded Samples for Next Generation Sequencing: Problems and Solutions
Source: Genes (Basel). 2021 Sep 23;12(10):1472. doi: 10.3390/genes12101472 (PMC8535326; doi:10.3390/genes12101472)
Supplement: Supplementary file 1 [file genes-12-01472-s001.zip › genes-1386375-supplementary.pdf]

Table S1. Most significant studies present in literature.

Number of References	Author(s)	Year	Topic(s)
[11]	Xuan, J. et Al.	2012	NGS and clinic
[12]	Do, H. et Al.	2015	NGS and artifact
[13]	Schweiger, M.R. et Al.	2009	NGS for FFPE
[17]	Ludyga, N. et Al.	2012	Artifact of sequence
[18]	Williams, C. et Al.	1999	Mutations in FFPE
[19]	Quach, N. et Al.	2004	Artifact and PCR
[20]	Ruiz, M.I.G. et Al.	2007	FFPE of NSCLC
[21]	Murray, S. et Al.	2008	Mutations in FFPE of NSCLC
[22]	Tsao, M. et Al.	2005	Erlotinib in NSCLC
[23]	Didelot, A. et Al.	2013	Multiplex digital PCR and FFPE
[24]	Suzuki, T. et Al.	1994	Artifacts in FFPE
[25]	Zsikla, V. et Al.	2004	Formalin and FFPE
[26]	Ofner, R. et Al.	2017	Artifacts in FFPE
